# Glycoprotein Profile Assessed by ^1^H-NMR as a Global Inflammation Marker in Patients with HIV Infection. A Prospective Study

**DOI:** 10.3390/jcm9051344

**Published:** 2020-05-04

**Authors:** Ana-Irene Malo, Anna Rull, Josefa Girona, Pere Domingo, Rocío Fuertes-Martín, Núria Amigó, Cèlia Rodríguez-Borjabad, Neus Martínez-Micaelo, Manuel Leal, Joaquim Peraire, Xavier Correig, Francesc Vidal, Lluis Masana

**Affiliations:** 1 Vascular Medicine and Metabolism Unit, Research Unit on Lipids and Atherosclerosis, Sant Joan University Hospital, Universitat Rovira i Virgili, IISPV, CIBERDEM, 43201 Reus, Spain; anairenemalo@gmail.com (A.-I.M.); josefa.girona@urv.cat (J.G.); celianutricio@gmail.com (C.R.-B.); neus.martinez@urv.cat (N.M.-M.); 2 INIM group and HIV Unit, Department of Internal Medicine and Infectious Diseases, Joan XXIII University Hospital, Universitat Rovira i Virgili, IISPV. 43005 Tarragona, Spain; anna.rull@iispv.cat (A.R.); jjperaire@gmail.com (J.P.); fvidalmarsal.hj23.ics@gencat.cat (F.V.); 3 Infectious Diseases Unit, Hospital de la Santa Creu i Sant Pau, 08041 Barcelona, Spain; pdomingo@santpau.cat; 4 Biosfer Teslab SL, DEEEA, Metabolomics Platform, Universitat Rovira i Virgili, IISPV, CIBERDEM, 43007 Tarragona, Spain; rociofrtsm@gmail.com (R.F.-M.); nuria.amigo@urv.cat (N.A.); 5 Internal Medicine Service, Viamed-Santa Ángela de la Cruz Hospital, 41014 Seville, Spain; mleal@telefonica.net; 6 Department of Electronic Engineering, Universitat Rovira i Virgili, IISPV, CIBERDEM, 43007 Tarragona, Spain; xavier.correig@urv.cat

**Keywords:** HIV-1, glycoprotein, proton nuclear magnetic resonance (^1^H-NMR), inflammation, immunological responders

## Abstract

Plasma glycoproteins are a composite biomarker of inflammation and can be detected by ^1^H-NMR. The aim of this study was to prospectively appraise the clinical value of plasma glycoproteins assessed by ^1^H-NMR in people living with HIV (PLWH). A total of 221 patients with HIV infection were recruited and studied at baseline and at 48 and 144 weeks. Patients were distributed into two groups according to baseline CD4^+^ T-cell number below or above 200 cells/µL. Patients with fewer than 200 cells/µL were distributed into the responders and nonresponders according to antiretroviral therapy (ART) response at 144 weeks. Glycoprotein concentrations were determined by ^1^H-NMR arising from the protein bond N-acetylglucosamine and N-acetylgalactosamine signals (GlycA); and N-acetylneuraminic acid signal (GlycB) associated with the sugar–protein bond concentration and aggregation state (shapes (height/width)). Basal glycoprotein concentrations were higher in patients with < 200 CD4^+^ T-cell/μL (Glyc A: 1040(917.9–1199.1) vs. 950.4(845.5–1050.9), *p* < 0.001, and Glyc B: 521(440.3–610.3) vs. 468.6(417.9–507.0) μ mol/L, *p* < 0.001) being reduced by ART. The height/width (H/W) ratio was the parameter showing a better association with this clinical status. Baseline glycoproteins predict the condition of responder/nonresponder. In this study, ^1^H-NMR glycoproteins provide novel insights to assess inflammation status and have prognostic value in PLWH.

## 1. Introduction

In recent decades, antiretroviral therapy (ART) has produced a drastic decrease in mortality related to immunosuppression in HIV-infected patients [1], exchanging a deadly disease for a chronic disease. This is due to the powerful effects that ART produces on viral suppression and immune restoration. In spite of this, ART does not completely restore immunity, particularly in patients presenting with advanced disease, instead maintaining a state of systemic dysfunctional activation of the immune system [2]. This state is associated with proinflammatory mediators, dysfunctional and senescent regulator T-cells, and a decrease in global immune competence, among others [3]. This low-degree proinflammatory state has a direct involvement in the pathogenesis and evolution of HIV infection. Moreover, it has been associated with several non-AIDS co-morbidities [3,4,5,6,7,8,9,10]. Several reports elsewhere have evidenced that subsequent non-AIDS events are more prevalent in patients who present with advanced disease who do not fully restore immunity despite appropriate viral suppression, the so-called immunological nonresponders (INRs) [11,12,13,14,15]. As an expression of the state of immune activation, different inflammatory markers have been studied, showing their relation to the appearance of morbidities/non-AIDS events and mortality [4]. Among these, the most frequently described are interleukin-6 (IL-6), D-Dimer [5,6,7,8,9], C-reactive protein (hsCRP) [10,11,12,13], fibrinogen, tumor necrosis factor (TNF), soluble CD14 levels (activation markers of monocytes and independent mortality predictor), and markers of intestinal barrier dysfunction and bacterial translocation (intestinal fatty acid binding protein (I-FABP), lipopolysaccharide (LPS), and bacterial DNA) [14,15,16,17]. However, the techniques used to measure a single inflammation biomarker such as hsCRP have sensitivity differences. They are associated with a single inflammation pathway and may be subject to variability between individuals and intraindividual fluctuations. Moreover, the correlation is not always present, and there is also no precise and standardized relationship between its plasma concentrations and the appearance of events [18]; therefore, searching for new systemic inflammation markers such as glycoproteins is warranted.

^1^H-NMR spectroscopy allows for rapid and accurate quantification of plasma glycosylated proteins from the quantification of the signal associated with the protons of the side chains of the N-acetyl carbohydrate groups (glucosamine, galactosamine and sialic acid), which are covalently linked to plasma proteins [19]. This technique allows the global identification of the glycoproteins involved in the inflammatory process. During the acute inflammatory phase of different processes, the concentration of glycosylated proteins increases, and their structure is modified by increasing the ramifications and number of monosaccharide residues. Unlike common biomarkers of inflammation, such as hsCRP or inflammatory cytokines, glycoprotein A (GlycA) and B (GlycB) are composite biomarkers that integrate the protein levels and glycation states of several of the most abundant acute-phase proteins in serum (α1-acid glycoprotein, haptoglobin, α1-antitrypsin, α1-antichymotrypsin, transferrin, and others) and play a key role in inflammatory processes [20,21]. Apart from their concentrations, other new variables, such as the parameters derived from the signal area and shape as the height and width of GlycA and GlycB, expressed as a ratio (H/W), are associated with the amount and the aggregation state or flexibility of the sugar–protein bonds generating glycosylation and have been related to inflammation status. Some investigations have demonstrated GlycA and GlycB to be good biomarkers of low-grade inflammation, and an association between these glycoproteins and several metabolic and cardiovascular events has been reported [19].

Because plasma glycoproteins play a crucial role in the modulation of the innate and adaptive immune systems, inflammation and pathological processes [20,22], they can be considered potential early diagnostic biomarkers of diseases associated with immune alterations, such as HIV infection [23,24].

In this work, we have studied plasma glycoprotein profiles assessed by ^1^H-NMR as marker of low-grade inflammation in patients with HIV infection and their correlation with clinical and standard biochemical parameters and, prospectively, their clinical prognosis value.

## 2. Materials and Methods

### 2.1. Study Design and Participants

**Figure 1:**
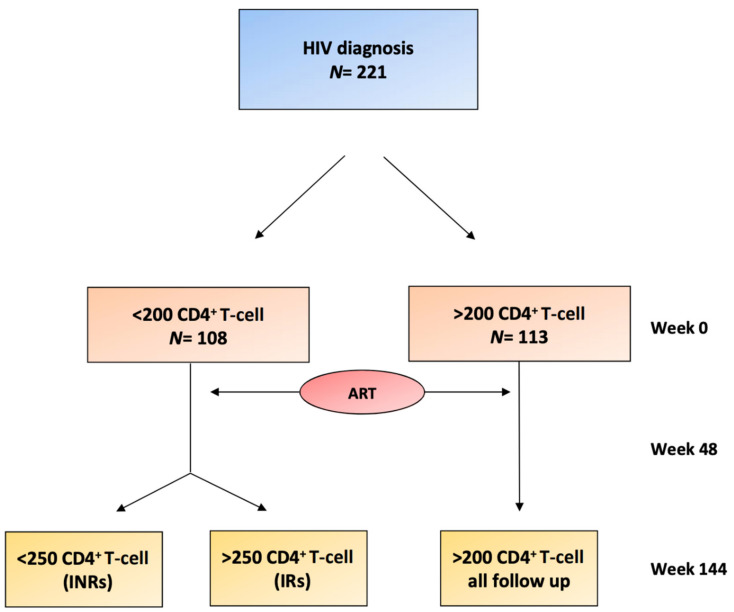
Flowchart of the patients included in the study. At week 0, the two groups were differentiated depending on the levels of CD4^+^ T-cells (<200 CD4^+^ T-cells or >200 CD4^+^ T-cells). Antiretroviral therapy (ART) therapy was initiated with subsequent follow-up at 48 weeks and at 144 weeks. At 144 weeks, two groups (IRs and INRs) were differentiated within the <200 CD4^+^ T-cell baseline group, depending on whether they achieved >250 CD4^+^ T-cells at week 144. INRs, immunological nonresponders; IRs, immunological responders.

This is a prospective, multicenter study. People living with HIV infection (PLWH) were recruited from three university hospitals (Hospital Joan XXIII, Tarragona, Hospital de la Santa Creu i Sant Pau, Barcelona, and Hospital Virgen del Rocío, Sevilla). A total of 221 adults with HIV infection (39 (33–45) years-old) who were ART naïve were recruited between 2009 and 2011 and were followed up on for up to 3 years. The exclusion criteria were the presence of opportunistic infections at the beginning of the study, active inflammatory diseases (except for diabetes mellitus, dyslipidemia or hepatitis coinfection), cancer or associated life-limiting diseases. The 221 patients included were studied at the basal timepoint (treatment naïve) and after 48 and 144 weeks of follow-up. Based on their naïve CD4^+^ T-cell count, we classified patients into two groups at baseline (<200 CD4^+^ T-cells and >200 CD4^+^ T-cells). At 144 weeks on ART, the group with <200 CD4^+^ T-cells at the basal timepoint was sorted into two subgroups: INRs if their CD4^+^ T-cell count was less than 250 cells/mL or immunological responders (IRs) if it was equal to or greater than 250 cells/mL (Figure 1). The prescribed ART at weeks 48 and 144 was also recorded. All centers obtained the approval of the ethical committee, and the participants signed informed consent forms.

### 2.2. Data Collection

At each study point, a complete physical examination was performed. Blood samples were obtained after an overnight fast. Plasma and serum aliquots were obtained and kept frozen at −80 °C in the Biobank of the Research Institute (IISPV) until used for analysis. HIV RNA viral load (VL), CD4^+^ T-cell count, and standard laboratory parameters (renal and liver function and lipid profile) were assessed. Therapy data were also recorded, and a standardized questionnaire was administered to evaluate adherence to ART [25].

### 2.3. Glycoprotein Analysis by ^1^H-NMR

Before ^1^H-NMR analysis, 200 μL of serum was diluted with 50 µL of deuterated water and 300 µL of 50 mM phosphate buffer solution (PBS) at pH 7.4. ^1^H-NMR spectra were recorded at 310 K on a Bruker Avance III 600 spectrometer operating at a proton frequency of 600.20 MHz (14.1 T) following previously reported procedures [26]. Briefly, we analyzed the region of the ^1^H-NMR spectrum where the glycoproteins resonate (2.15–1.90 ppm) using several functions according to the chemical shift: GlycF, GlycA and GlycB. For each function, we determined the total area and transformed it to concentration according to the number of sugar–protein bonds. The area, height, position, and bandwidth and their ratios were also calculated. The concentrations of GlycA and GlycB provided the amount of acetyl groups of protein bond N-acetylglucosamine, N-acetylgalactosamine, and N-acetylneuraminic acid. The GlycF area arises from the concentration of −COCH3 acetyl groups of N-acetylglucosamine, N-acetylgalactosamine, and N-acetylneuraminic unbonded to proteins (free fraction). Height/width ratios of GlycA and GlycB (H/W) were associated with the shape of the peaks generating the ^1^H-NMR signals indicating the aggregation state or flexibility of the sugar–protein bonds [26,27].

### 2.4. Statistical Analysis

Continuous variables were tested for normality using the Kolmogorov–Smirnov test. Data are presented as the medians and 25th and 75th percentiles for continuous variables not normally distributed or the mean and standard deviation (SD) when normally distributed. Categorical variables are expressed as percentages unless otherwise indicated. Differences between groups (<200 CD4^+^ T-cells, >200 CD4^+^ T-cells) were analyzed by the nonparametric Mann–Whitney test or parametric Student’s *t*-test for continuous variables and the chi-squared test for categorical variables. Adjusted differences were investigated using analyses of covariance (ANCOVAs). Associations between glycoproteins and continuous variables were analyzed by Spearman’s correlation test. Random forest analysis was performed to provide a selection of glycoproteins that were better classified between groups (<200 CD4^+^ T-cells, >200 CD4^+^ T-cells). Logistic regression models and receiver-operating characteristic curves (ROC) were also performed to assess any predictive value to identify IRs and INRs patients at week 144. Statistical analyses were conducted with the SPSS 25 package (IBM, Madrid, Spain). A *p* value less than 0.05 was considered statistically significant.

## 3. Results

### 3.1. Baseline Clinical and Analytical Parameter Data

At baseline, 221 HIV antiretroviral drug-naïve patients who met the inclusion criteria were classified into two groups: 108 (48.86%) were classified in the <200 CD4^+^ T-cells/µL group and 113 (51.13%) in the >200 CD4^+^ T-cells/µL group. Both groups were similar with respect to sex distribution, type 2 diabetes, dyslipidemia, and renal dysfunction. The CD4^+^ T-cell <200/µL group tended to be older than the CD4^+^ T-cell >200/µL group. There were also differences in gamma-glutamyl-transferase (GGT) and alkaline phosphatase (FA) without differences in alanine aminotransferase (ALT) and aspartate aminotransferase (AST) between groups. No differences in hepatitis co-infection was observed. As expected, the viral load was also different between groups (Table 1).

### 3.2. Associations of Glycoproteins with Analytical Parameters at the Basal Timepoint

Higher levels of glycoproteins at baseline were significantly associated with the <200 CD4^+^ T-cells/µL group in comparison with the >200 CD4^+^ T-cells/µL group. Differences in the concentrations and H/W ratios of the glycoproteins remained significant after adjusting for age, FA, GGT, and VL (Table 1). Random forest analysis revealed that baseline H/W GlycA and H/W GlycB were the parameters that best classified the patients in these two groups (Supplemental Materials Figure S1).

HsCRP concentrations were higher in the <200 CD4^+^ T-cells/µL group (Table 1). Baseline GlycA and B concentrations and the H/W ratios were correlated with hsCRP values (Table 2).

At baseline, glycoproteins showed a significant inverse association with baseline CD4^+^ T-cell levels and a positive relationship with VL (Table 2). From a metabolic point of view, we found an association between glycoprotein levels and triglycerides and an inverse correlation with HDL cholesterol. After adjusting for age and viral load, the GlycA and GlycB concentrations and H/W ratios maintained significant correlations with HDL-C, CD4^+^ T-cells, and hsCRP. GlycA and its H/W ratio also maintained their significant correlation with triglycerides after adjustment (Table 2).

### 3.3. Evolution of Glycoproteins and Prognosis in the Prospective Study

The basal concentrations of GlycA and GlycB and their H/W ratios in the <200 CD4^+^ T-cells/µL group were similar between IRs and INRs at week 144. However, they were correlated with the final CD4^+^ T-cell count (week 144) when analyzing all patients (Supplemental Materials Table S1). This correlation was not found when performing analysis by groups (CD4^+^ T-cells <200 vs. CD4^+^ T-cells > 200) or by subgroups (IRs vs. INRs), attributable to the decrease in sample size.

**Figure 2:**
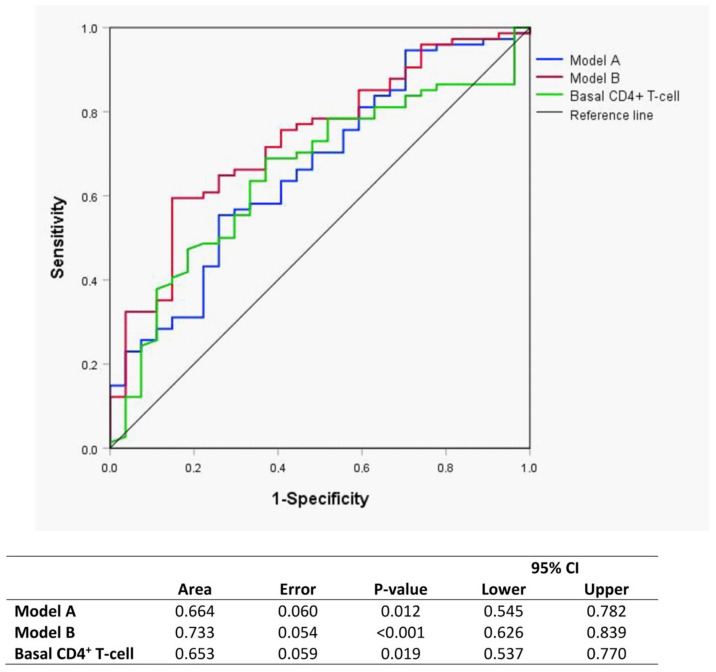
Receiver-operating characteristic curves (ROC) curves of Model A (blue) and Model B (red) for evaluating glycoprotein biomarkers as predictors of the immunological response to ART at baseline. Model A: Area GlycA, Area GlycB, height/width (H/W) GlycA and H/W GlycB. Model B: Model A, basal CD4^+^ T-cell count and age.

In the <200 CD4^+^ T-cells/µL group, the ROC curves show that the model (Figure 2, model A) that contained each basal glycoprotein concentration and ratio defined an area under the curve of 0.664 (*p* = 0.012) for the prediction of which patients will be IRs or INRs at week 144. The model with all glycoproteins by ^1^H-NMR was a better predictor of immune response than the baseline CD4^+^ T-cell level (area under the curve 0.653, *p* = 0.019). With these results, we generated a model (Figure 2, model B) using model A, baseline CD4^+^ T-cell level and age. This model predicted the condition of immune responder/nonresponder with an area of 0.733 (*p* < 0.001) for early identification even before the start of ART treatment, which was better than basal CD4^+^ T-cells alone. There were no differences in ART between IRs and INRs.

**Figure 3:**
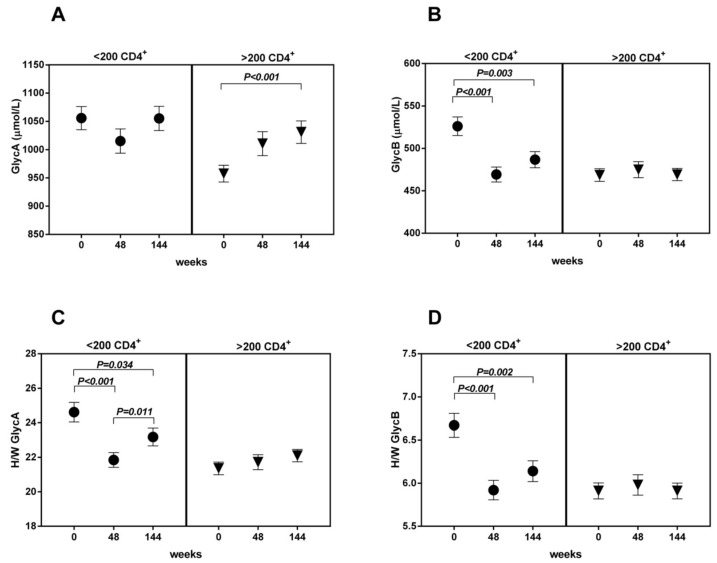
The evolution of glycoproteins ((**A**): Glycoprotein A; (**B**): Glycoprotein B; (**C**,**D**) Height/Width ratio for glycoprotein A and B respectively) is represented according to groups (basal <200 CD4^+^ T-cells vs. >200 basal CD4^+^ T-cells) and time (weeks 0–48–144).

**Figure 4:**
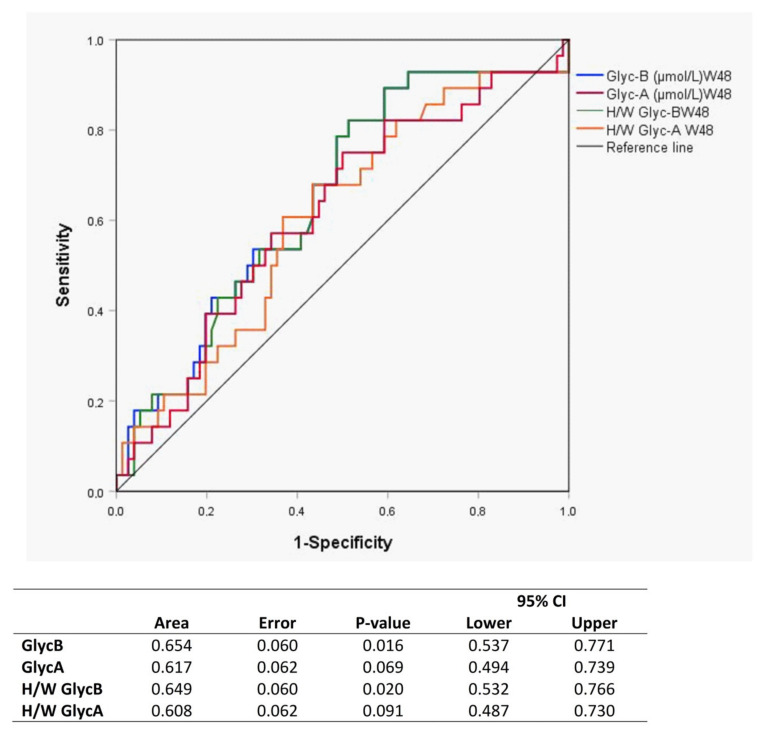
ROC curves of each glycoprotein as a predictor biomarker of immunological response to ART at week 48.

In the <200 CD4^+^ T-cells/µL group, the GlycB concentrations and H/W ratios showed a significant decrease at week 48, and the level was maintained up to week 144 (Figure 3B,D). The GlycA evolution showed a similar trend, although only the H/W ratio achieved statistical significance (Figure 3A,C). This pattern was not observed in the >200 CD4^+^ T-cells/µL group. The ROC curves including GlycB and its H/W ratio separately at week 48 show a predictive value of immunological response (Figure 4).

## 4. Discussion

Here, we studied the plasma glycoprotein profile assessed by ^1^H-NMR in PLWH. ^1^H-NMR allows measurement of the global plasma sugar–protein bond concentration of glycoproteins, referred to as GlycA and GlycB, as well as some related parameters such as the aggregation state (or flexibility) of the sugar–protein bonds. These data are provided by the shape of the NMR signal associated with plasmatic glycoproteins derived from the ratio between its height and width (H/W). The higher the ratio, the greater is the inflammation state, and indicates a more flexible—and probably more accessible—bond between the sugar and the glycosylated protein. Given that low-grade inflammation is a hallmark finding in treated PLWH, even in those who achieve virologic suppression, and that this low-grade inflammatory state has been linked with several non-AIDS conditions, is seems worth investigating glycoproteins as a potential biomarker of inflammation in this clinical setting.

We have observed that GlycA and GlycB concentrations are higher in patients with a worse immunological state and are also an indicator of prognosis. Those patients with higher baseline glycoprotein concentrations tend to respond less to ART. The H/W ratio is also directly associated with the clinical state and prognosis of patients.

The rationale behind these observations is that during acute phase inflammatory states, the concentration of glycosylated proteins increases, and their structure is modified by increasing the ramifications and number of monosaccharide residues, such as N-acetylglucosamine, N-acetylgalactosamine (GlycA) or N-acetylneuraminic acid (GlycB), among others, playing a key role in inflammatory processes [19,20]. These glycoproteins are a marker composed of different acute phase proteins and their glycation states (α1 acid glycoprotein, haptoglobin, α1 antitrypsin, α1 antichymotrypsin and transferrin), representing a more global measure and therefore more stable inflammatory status [28] than traditional markers such as hsCRP. On the other hand, these parameters show less intravariability between individuals [29,30].

The composite nature of ^1^H-NMR glycoprotein measurement makes it possible to better capture systemic inflammation, with a greater association with morbidity and mortality compared to single biomarkers such as hsCRP [31,32,33].

In the HIV population, changes in glycoprotein concentrations have been evaluated based on the antiretroviral therapy used, with a decrease seen in GlycA both in patients undergoing treatment with integrase inhibitors (raltegravir) and ritonavir-boosted protease inhibitors [34]. Another study evaluated the association of GlycA in HIV patients with the presence of subclinical coronary arteriosclerosis, suggesting that this new marker could stratify cardiovascular risk in this population [35]. As far as we know, there are no studies in the literature that evaluate glycoproteins as prognostic markers for the condition of immunological responders. In a previous study in the same cohort, metabolic differences in the lipoprotein profile measured by ^1^H-NMR were found between responding and nonresponding patients at baseline [36].

The CD4^+^ T-cell count at diagnosis [37], age, parenteral drug use [38], and the rapid decrease in CD4^+^ T-cells [39] are factors related to poor prognosis and reaching an optimal CD4^+^ T-cell level. Basal glycoprotein values were correlated to both CD4^+^ T-cells and viral load. Viral replication occurring under nonsuppressive therapy could lead to inflammation, jeopardizing the relevance of our results; therefore, all data were adjusted for these variables. Although the baseline determination of a single glycoprotein group by itself did not predict the immunological recovery, the compilation of data related to glycoprotein measurement obtained by the ^1^H-NMR methods (glycoprotein groups A and B plus H/W ratios) did predict the recovery condition at baseline. The joint determination of glycoproteins even before the start of treatment was superior to the baseline CD4^+^ T-cell count for the prediction of IRs or INRs. A higher prediction for this condition was found by including baseline glycoprotein, CD4^+^ T-cells + levels and age together in a model, thus increasing the predictive value with respect to CD4^+^ T-cell levels alone. Moreover, baseline glycoprotein levels were correlated with the final CD4^+^ T-cells/µL count in the whole group.

Another interesting observation was the impact of therapy on inflammation markers. The striking reduction in glycoproteins at week 48 of treatment with respect to the baseline supports the early onset of antiretroviral therapy in infected patients.

Limitations of our study are the sample size, but taking into account the follow-up period results in a very informative cohort. HsCRP was available in a limited number of patients precluding head-to-head comparisons with glycoproteins but providing enough robustness to obtain reliable data at basal levels and correlations to glycoproteins. Data on obesity, a possible confounding factor because it is associated to chronic low inflammation degree, were incomplete and thus could not be analyzed, although in this group of patients, a significant prevalence of obesity was unexpected. The information given by the ROC curves performed to assess the predictive value of glycoproteins, although widely used in biomedicine, could be improved by other statistical techniques that were unavailable, such as total operating characteristic (TOC) curves that provide a broader information for each predictive threshold [40]. Glycoprotein profiling by ^1^H-NMR spectroscopy is a currently developing method, and technical improvements can be expected, although no significant changes in results are envisaged.

## 5. Conclusions

Plasma NMR spectroscopy allows a better understanding of metabolic changes in complex diseases. PLWHs are exposed lifelong to alterations in the immune system, including inflammation mechanisms, leading to complications not directly associated with HIV. According to our results, NMR glycosylation profiling focused on inflammation mediators could contribute to a better knowledge of these chronic conditions. GlycA and B signal characterization provide a wider view of inflammatory status than concentrations of a single biomarker, increasing the robustness of inflammation assessment.

## Figures and Tables

**Table 1:** Clinical and analytical characteristics of the study subjects. ^1^H-NMR glycoproteins at baseline.

	All, *n* = 221	<200 CD4^+^ T-Cell, *n* = 108	>200 CD4^+^ T-Cell, *n* = 113	*p* Value
Clinical characteristics				
Age (y)	39 (33–45)	40 (34–48)	37 (30–43)	0.009
Gender (%, women)	39 (17.6)	20 (18.5)	19 (16.8)	0.890
Type 2 Diabetes (%, yes)	16 (7.3)	6 (5.6)	10 (8.9)	0.439
Hepatitis (%, yes)	103 (47.5)	52 (49.1)	51 (45.9)	0.684
Biochemical parameters				
Cholesterol (mmol/L)	4.2 ± 1	4.7 ± 1.2	4.3 ± 0.8	0.422
Triglycerides (mmol/L)	1.3 (0.9–1.9)	1.39 (1–2.0)	1.2 (0.9–1.8)	0.113
LDL-C (mmol/L)	2.5 ± 0.9	2.5 ± 1.1	2.6 ± 0.6	0.788
HDL-C (mmol/L)	0.9 (0.8–1.1)	0.9 (0.7–1.1)	1(0.8–1.1)	0.841
AST (µkat/L)	0.7 (0.5–0.9)	0.7 (0.6–1)	0.7 (0.5–1)	0.158
ALT (µkat/L)	0.6 (0.5–1)	0.6 (0.5–1.1)	0.6 (0.5–0.9)	0.491
GGT (µkat/L)	0.8 (0.4–1.5)	1 (0.6–2.1)	0.5 (0.4–1.0)	<0.001
ALP (µkat/L)	0.7 (0.6–0.9)	0.8 (0.6–1)	0.6 (0.5–0.8)	<0.001
Creatinine (µmol/L)	91 (82–100)	90 (79–97)	92 (86–103.5)	0.090
GF (mL/min/1.73 m^2^)	87 (77–101)	87 (79–105)	88 (77–100)	0.717
CD4^+^ T-cell (cells/µL)	223 (103–331)	92 (34–177)	328 (272–441)	<0.001
VL (log copies/mL)	4.98 (4.52–5.49)	5.24 (4.68–5.56)	4.77 (4.32–5.13)	<0.001
HsCRP * (mg/L)	1.6(0.70–9.00)	2.1 (0.8–10)	1.3 (0.4–5.5)	0.036
Glycoproteins				
Glyc B (µmol/L)	489.1 (431.4–545.7)	521 (440.3–610.3)	468.6 (417.9–507.0)	<0.001
Glyc A (µmol/L)	972.5 (890.1–1120.0)	1040 (917.9–1199.1)	950.4 (845.5–1050.9)	<0.001
H/W Glyc B	6.2 (5.5–7)	6.6 (5.6–7.8)	5.9 (5.3–6.9)	<0.001
H/W Glyc A	21.9 (19.9–25.77)	24.3 (20.6–29.33)	21.3 (19.44–23.22)	<0.001
Treatment during follow-up				
PI 48 weeks (%)	115 (52.0)	59 (54.6)	56 (49.6)	0.501
TDF 48 weeks (%)	83 (37.6)	46 (42.6)	37 (32.7)	0.165
PI 144 weeks (%)	95 (43.0)	51 (47.2)	44 (38.9)	0.224
TDF 144 weeks (%)	81 (36.7)	47 (43.5)	34 (30.1)	0.050

Data are shown as the *n* (percentage) for qualitative variables and median (25th percentile–75th percentile) or mean ± SD for quantitative variables. *P* values for group comparisons are reported. * Hs-CRP *n* = 124. For the group <200 CD4^+^ T-cells, *n* = 68. For the group >200 CD4^+^ T-cells, *n* = 56. LDL-C, low-density lipoprotein–cholesterol; HDL-C, high-density lipoprotein–cholesterol; AST, aspartate aminotransferase; ALT, alanine aminotransferase; GGT, gamma-glutamyl transferase; ALP, alkaline phosphatase; GF, glomerular filtration; VL, viral load; hsCRP, high-sensitivity C-reactive protein; PI, protease inhibitors; TDF, tenofovir.

**Table 2:** Associations between ^1^H-NMR glycoprotein variables and biochemical characteristics of the study subjects at baseline.

	GlycB	GlycA	H/W GlycB	H/W GlycA
Age	0.090	0.198 **	0.084	0.128
Cholesterol	−0.019	0.134	−0.018	0.017
Triglycerides	0.221 **	0.353 **	0.221 **	0.236 **
LDL-C	0.024	0.165	0.026	0.063
HDL-C	−0.259 **	−0.226 **	−0.263 **	−0.251 **
GOT	−0.103	−0.095	−0.093	−0.048
GPT	−0.132	−0.067	−0.111	−0.093
GGT	0.063	0.057	0.072	0.142
FA	0.180 *	0.116	0.175 *	0.210 **
Creatinine	−0.050	−0.035	−0.056	−0.082
GF	0.034	-0.032	0.034	0.007
CD4^+^ T-cell	−0.337 **	−0.297 **	−0.350 **	−0.381 **
VL	0.098	0.092	0.120	0.221 **
Hs-CRP *	0.456 **	0.454 **	0.458 **	0.512 **

Spearman correlation coefficients and *p* values (* *p* < 0.05. ** *p* < 0.001) for each glycoprotein variable and biochemical parameter. LDL-c, low-density lipoprotein–cholesterol; HDL-c, high-density lipoprotein–cholesterol; AST, aspartate aminotransferase; ALT, alanine aminotransferase; GGT, gamma-glutamyl transferase; ALP, alkaline phosphatase; GF, glomerular filtration; VL, viral load; hsCRP, high-sensitivity C-reactive protein. *n* = 221, * *n* = 121.

## Supplementary Materials

The following are available online at https://www.mdpi.com/2077-0383/9/5/1344/s1, Figure S1: Random forest analysis, Table S1: Spearman correlation coefficients between basal glycoproteins and final CD4^+^ T-cells (144 weeks).
