# Supplementary material for: Glycoprotein Profile Assessed by 1H-NMR as a Global Inflammation Marker in Patients with HIV Infection. A Prospective Study
Source: J Clin Med. 2020 May 4;9(5):1344. doi: 10.3390/jcm9051344 (PMC7291035; doi:10.3390/jcm9051344)

Supplemental Table

Table S1. Spearman correlation coefficients between basal glycoproteins and final CD4$^+$ T-cells (144 weeks).

	CD4$^+$ T-cell	
	r	*P* value
GlycB	- 0.208	0.003
GlycA	-0.173	0.013
H/W GlycB	-0.209	0.003
H/W GlycA	-0.207	0.003

r, Spearman correlation coefficient.

Supplementary Figure

Figure S1. Random forest analysis. The concentrations and the H/W ratios of GlycA and GlycB in the three stages (week 0, week 48 and week 144) were analyzed. The H/W ratios of GlycA and GlycB at week 0 are the parameters that best differentiated the two basal groups (<200 CD4$^+$ T-cells/μL vs.> 200 CD4$^+$ T-cells/μL).

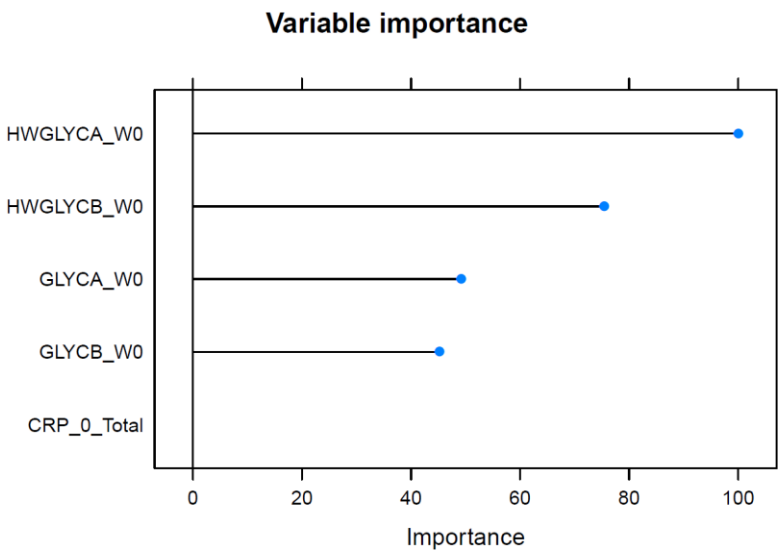

Supplement: Supplementary file 1 [file jcm-09-01344-s001.pdf]
